# Machine learning approach to monitor inkjet jetting status based on the piezo self-sensing

**DOI:** 10.1038/s41598-023-45445-0

**Published:** 2023-10-23

**Authors:** Thanh Huy Phung, Sang Hyeon Park, Inyoung Kim, Taik-Min Lee, Kye-Si Kwon

**Affiliations:** 1 Department of Mechatronics, Ho Chi Minh City University of Technology (HCMUT), 268 Ly Thuong Kiet Street, District 10, Ho Chi Minh City, 700000 Vietnam; 2 Vietnam National University Ho Chi Minh City (VNU-HCM), Linh Trung Ward, Thu Duc, Ho Chi Minh City, 700000 Vietnam; 3 Department of Electronic Materials Devices and Equipment Engineering, Soonchunhyang University, 22 Soonchunhyang-ro, Shinchang, Asan-si, Chungnam 31538 Republic of Korea; 4 Department of Flexible and Printed Electronics, Korea Institute of Machinery and Materials (KIMM), 156 Gajeongbuk-ro, Yuseong-gu, Daejeon, 34103 Republic of Korea; 5 Department of Robot and Manufacturing System, Korea University of Science and Technology (UST), 217 Gajeong-ro, Yuseong-gu, Daejeon, 34113 Republic of Korea; 6 Department of Mechanical Engineering, Soonchunhyang University, 22 Soonchunhyang-ro, Shinchang, Asan-si, Chungnam 31538 Republic of Korea

**Keywords:** Electrical and electronic engineering, Mechanical engineering

## Abstract

One of the advantages of inkjet printing in digital manufacturing is the ability to use multiple nozzles simultaneously to improve the productivity of the processes. However, the use of multiple nozzles makes inkjet status monitoring more difficult. The jetting nozzles must be carefully selected to ensure the quality of printed products, which is challenging for most inkjet processes that use multi-nozzles. In this article, we improved inkjet print head monitoring based on self-sensing signals by using machine learning algorithms. Specifically, supervised machine learning models were used to classify nozzle jetting conditions. For this purpose, the self-sensing signals were acquired, and the feature information was extracted for training. A vision algorithm was developed to label the nozzle status for classification. The trained models showed that the classification accuracy is higher than 99.6% when self-sensing signals are used for monitoring. We also proposed a so-called hybrid monitoring method using trained machine learning models, which divides the feature space into three regions based on predicted jetting probability: certain jetting, certain non-jetting, and doubt regions. Then, the nozzles with uncertain status in the doubt region can be verified by jet visualization to improve the accuracy and efficiency of the monitoring process.

## Introduction

The application of inkjet printing has been expanding in the field of digital fabrication^1^. In particular, inkjet technology has been successfully used for various applications, such as 3D bioprinting^2–4^, sensors^5–9^, or energy devices^10–12^. In particular, inkjet contributes significantly to display manufacturing industries, including LCD, OLED, AMOLED, and micro-LED^13–19^. A major advantage of inkjet for industrial applications is that multi-nozzle, multi-array printheads can be used to significantly increase fabrication efficiency^1,20,21^. In such cases, monitoring the jetting condition of entire nozzles becomes important. If defective nozzles are selected for printing or the jetting status varies during the printing process, they can cause problems with printed products. For example, in the case of display manufacturing, dead pixels can be found in the final stage, and it could be costly for product inspection and disposal of malfunctioned products.

To verify the jetting condition and eliminate defective nozzles, jet visualization (visualize the jetting using high-speed cameras or stroboscopic techniques) and inspection of printed patterns (print the patterns and check whether the nozzles are jettable or not) are the two main approaches^1^, which are the basics for the development of different methods and technologies. However, real-time jet monitoring becomes more challenging as the number of nozzles for printing increases. For example, a jet visualization takes at least a second to visualize a droplet jetting from an individual nozzle, and a printhead with 1000 nozzles takes over 16 min to scan entire nozzles. Also, note that nozzle failures could occur during printing. Hence, fast real-time monitoring is required without mechanical alignment to cameras or sensors. To implement real-time monitoring, self-sensing techniques based on sensing the signals of piezoelectric nozzles have been introduced^22–25^.

In piezo-based inkjet printing, piezo actuators are used as nozzles for jetting. When the jetting driver applies waveforms to the nozzles, pressure waves are created, pushing the ink out of the nozzle and producing droplets^26^. After jetting, the residual pressure waves during jetting can influence back to the piezo actuators, causing piezo actuators to generate signals that can be sensed via the jetting drivers, so-called self-sensing signals. Here, the sensed signals are created during the jetting processes and can be used to monitor the jetting status of the nozzles. Because the self-sensing signals are acquired directly from the nozzles when the jetting waveform is applied, it becomes possible to achieve scanning frequencies (speeds) as high as tens of kilohertz. Furthermore, through the implementation of parallel scanning methods^22,25^, it is feasible to simultaneously sense multiple nozzles, thereby reducing the monitoring time for thousands of nozzles to mere seconds.

To monitor the nozzles using self-sensing, the sensed signals should be analyzed to extract features for the classification of nozzle jetting status. However, the decision criteria (threshold values) for the extracted features have been intuitively decided based on observations, which may not be optimal to maximize monitoring performance. Recently, S.H. Park and K.S. Kwon proposed the use of a statistical method to improve the decision-making process through scatter plots of a large number of data^25^. However, the approach was also based on observations, and further algorithm improvement is necessary. In this paper, we propose the use of machine learning to classify the jetting status of the nozzles, which can improve the decision criteria for selecting good jetting nozzles for printing.

Recently, efforts have been made to apply artificial intelligence (AI) and machine learning (ML) techniques to manufacturing, especially in the field of additive manufacturing^27–30^. Note that ML is considered to be a subset of AI techniques. The use of ML and AI techniques is expected to make the manufacturing process more intelligent. In inkjet printing, AI and ML have been also applied to improve the printing process. However, the main focus has been on understanding jetting^31^, selection^32^, and visualization-based jetting detection^33,34^, which are mainly based on drop visualization by scanning whole nozzles or specific target nozzles. However, utilizing visualized jet images for AI and ML applications in the detection of whole nozzle jet failures carries inherent limitations when applied to inkjet monitoring, primarily due to the lengthy scanning time required, making it unsuitable for real-time applications. To overcome the previous problems, this study proposes the utilization of machine learning as a tool for detecting misfiring nozzles based on piezo self-sensing. According to the authors’ best knowledge, this work is the first published work that uses modern ML techniques to determine nozzle jetting status for multi-nozzle inkjet printhead based on self-sensing signals.

In our methodology, we have customized machine learning algorithms to discern between "normal-jetting" (or simply "jetting", where nozzles consistently maintain a jet status aligned with a reference status) and "non-jetting" (where there are significant deviations from the reference). This classification ensures the optimal selection of nozzles for the printing process. It's worth noting that nozzles may encounter other types of failures, such as misdirection or slow jetting. However, in this study, our specific focus is on the non-jetting case, as it represents a significant deviation from the reference status and has a serious impact on the printing process. Also, we presume that jetting parameters, such as ink or waveforms, are already ideally adjusted for specific applications. The primary emphasis is on nozzle jetting quality rather than the morphology of the printed pattern. Printed morphology is influenced by factors such as the drying and spreading of droplets, which are distinct from jetting and non-jetting issues.

To determine the jetting status, we extracted two features, namely, phase and magnitude differences, from the self-sensing signals of the nozzles, which contain jetting information. Then, the extracted features were used as inputs to train the models (classifiers) to separate jetting nozzles from the non-jetting ones.

Additionally, an improved image processing method was proposed to analyze jetting images. This image processing method was used to label the jetting and non-jetting status for the self-sensing signals. The proposed ML models showed more than 99.6% accuracy in classifying the jetting status of each nozzle when the prepared data were used for verification.

Finally, we have developed a 'hybrid monitoring' methodology that integrates self-sensing based ML models with jet visualization. This approach employs prediction probability thresholds to determine whether a nozzle is jetting, non-jetting, or requires additional visualization for confirmation. In this way, the overall performance of the monitoring process can be enhanced.

The following sections include the proposed methodology, monitoring system and experiment setup, visualization and image processing algorithm, self-sensing signal acquisition and processing, and modeling of nozzle jetting status using machine learning approaches. Later, the results of the approaches are presented and discussed for possible use in industrial purposes.

## Methodology

### Monitoring system and experiment setup

**Figure 1:**
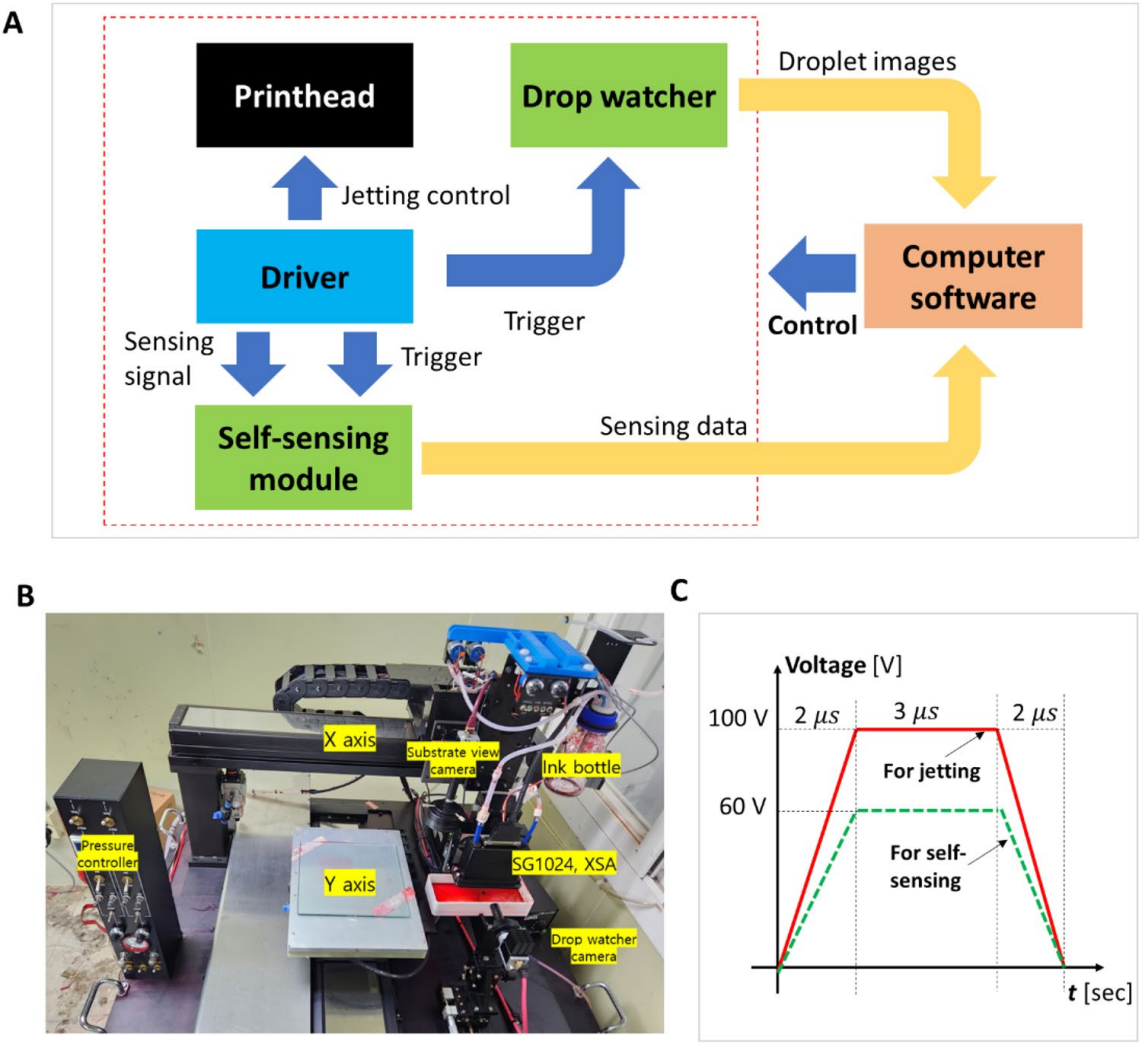
Jet monitoring system for multi-nozzle printhead inkjet. (**A**) Hybrid jet monitoring system capable of jet visualization and self-sensing, (**B**) photo of the implemented system, and (**C**) waveforms for jetting and sensing.

This study used a printing system with self-sensing and drop-watching capabilities for jet monitoring (Fig. 1). Figure 1A shows the overview of the printing and monitoring system. In the system, a printhead with 1024 nozzles (SG1024XSA, Fujifilm Dimatix, USA) was used for printing and examination. The printhead consisted of 8 rows with 128 nozzles per row. Each row has an independent driver, which controls the jetting of each nozzle. A drop visualization system was developed to inspect the nozzles through the visualized droplet images, and an in-house developed self-sensing module was used to scan and collect the self-sensing signals of the entire nozzles. As shown in Fig. 1A, self-sensing data acquisition and jet visualization are triggered by the jetting trigger signals from the inkjet head driver. An in-house software was developed to control the entire system, including jetting, printing patterns, and monitoring. By using the developed system and software, the signals and images of nozzle jetting were collected and saved for further processes. Figure 1B illustrates the implementation of the system.

In the experiments, the model fluid from the printhead manufacturer (XL-30, Fujifilm Dimatix, USA) was used for the jetting experiment, and the printhead temperature was maintained at 30 °C to stabilize the ink conditions. Ink can be one of the important parameters as it can affect the jetting characteristics. However, we do not consider other ink properties to focus on algorithms and methodologies without loss of generality. Here, two different trapezoidal waveforms (described in Fig. 1C) were used for jetting (100 V) and sensing (60 V). Note that using a lower voltage (60 V) for sensing has advantages since actual jetting is not required to monitor the jetting status.

### Machine learning modeling to classify nozzle jetting status

**Figure 2:**
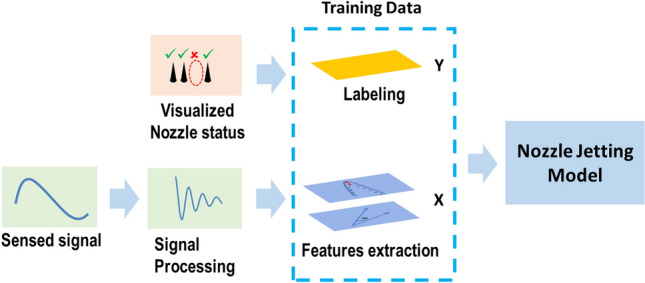
Modeling process for nozzle jetting classification.

In this study, machine learning models have been developed and customized to classify the nozzle jetting status by using the acquired self-sensing signals, which were labeled (jetting or non-jetting status) by the jetting image analysis as shown in Fig. 2. For this purpose, more than 150,000 jetting samples were collected. The labeling, data processing, and training processes are described in the following sections. After training, the models can be used to monitor the jetting status as well as to select the jetting nozzles for printing.

### Jet visualization and gradient-based image processing for labeling of nozzles according to jetting status

**Figure 3:**
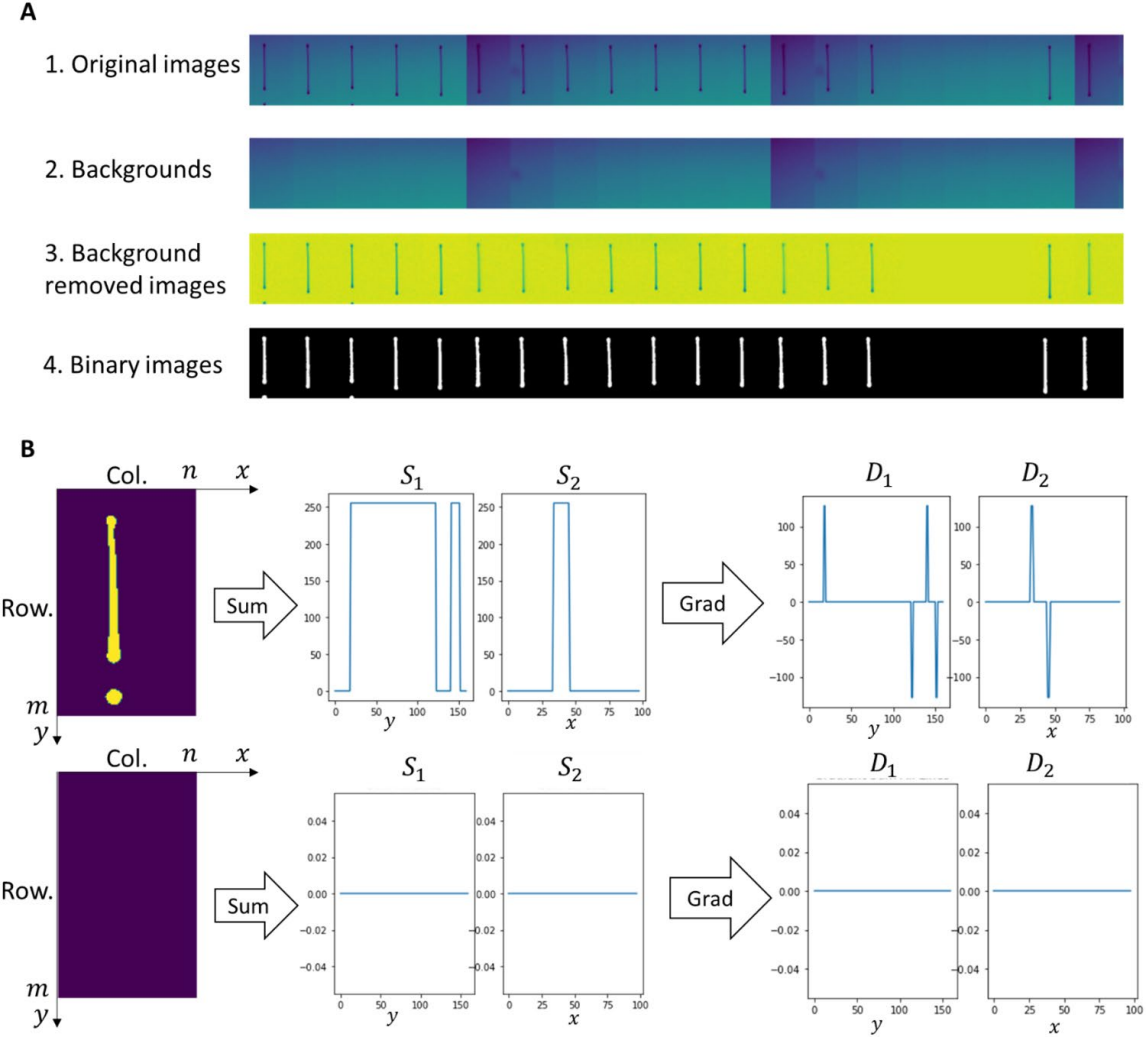
Image analysis method to determine the jetting status. (**A**) Pre-processing of images to obtain binary images, and (**B**) The pixel-gradient method to determine jetting status.

A CCD camera (Basler, acA1300-60gm, Germany) and a 2 × telecentric lens (TL20-65, Myutron, Japan) were used for jet visualization. The strobed illumination with respect to the jetting signals was used to acquire frozen jetting images from the camera. An imaging analysis algorithm was developed to determine the jetting status and label the sensing signal for the modeling process, as shown in Fig. 3.

To obtain accurate labels for each self-sensing signal (jetting status), images of nozzle jetting and their corresponding sensing signals were acquired almost simultaneously. Note that the obtained droplet images had different brightness levels for different locations of the jetting nozzles. It can be difficult to analyze a large number of images automatically when there are differences in brightness and contrast. To suppress those effects, the backgrounds (images acquired at the nozzle locations without jetting) (Fig. 3A.2) were subtracted from the original images, and the images were filtered to achieve uniform brightness (Fig. 3A.3). Finally, the images were converted to binary form (Fig. 3A.4) for further processing.

After pre-processing to get the binary jet images, the nozzle status was determined as illustrated in Fig. 3B. Consider each image to be an $I\left(x,y\right)$ matrix with a dimension of $m$ rows $\times$ $n$ columns:

1 $$I\left(x,y\right)=\left\{\begin{array}{c}0-dark \\ 255-brigth\end{array}\right..$$

Here, 0 represents the background, and 255 represents the value of the droplet images. Then, define the sums by rows, ${S}_{1}$, and columns, ${S}_{2}$, as:

2 $$\left\{\begin{array}{c}{S}_{1}\left(y\right)=\sum_{x=0}^{n-1}I(x,y)\\ {S}_{2}\left(x\right)=\sum_{y=0}^{m-1}I\left(x,y\right).\end{array}\right.$$

And the corresponding gradients:

3 $$\left\{\begin{array}{c}{D}_{1}\left(y\right)=\frac{\partial {S}_{1}}{\partial y}\\ {D}_{2}\left(x\right)=\frac{\partial {S}_{2}}{\partial x}\end{array}\right..$$

The illustration of ${S}_{1},{S}_{2},{D}_{1},$ and ${D}_{2}$ are shown in Fig. 3B. Note that the brightness values saturate at 0 and 255, and the values of ${S}_{1}$ and ${S}_{2}$ are only 0 or 255. As a result, the gradients had peaks as shown in Fig. 3B. Those peaks could be used to determine almost possible jetting nozzle states (Supplementary Information Sect. S1).

### Self-sensing data acquisition and feature extraction

**Figure 4:**
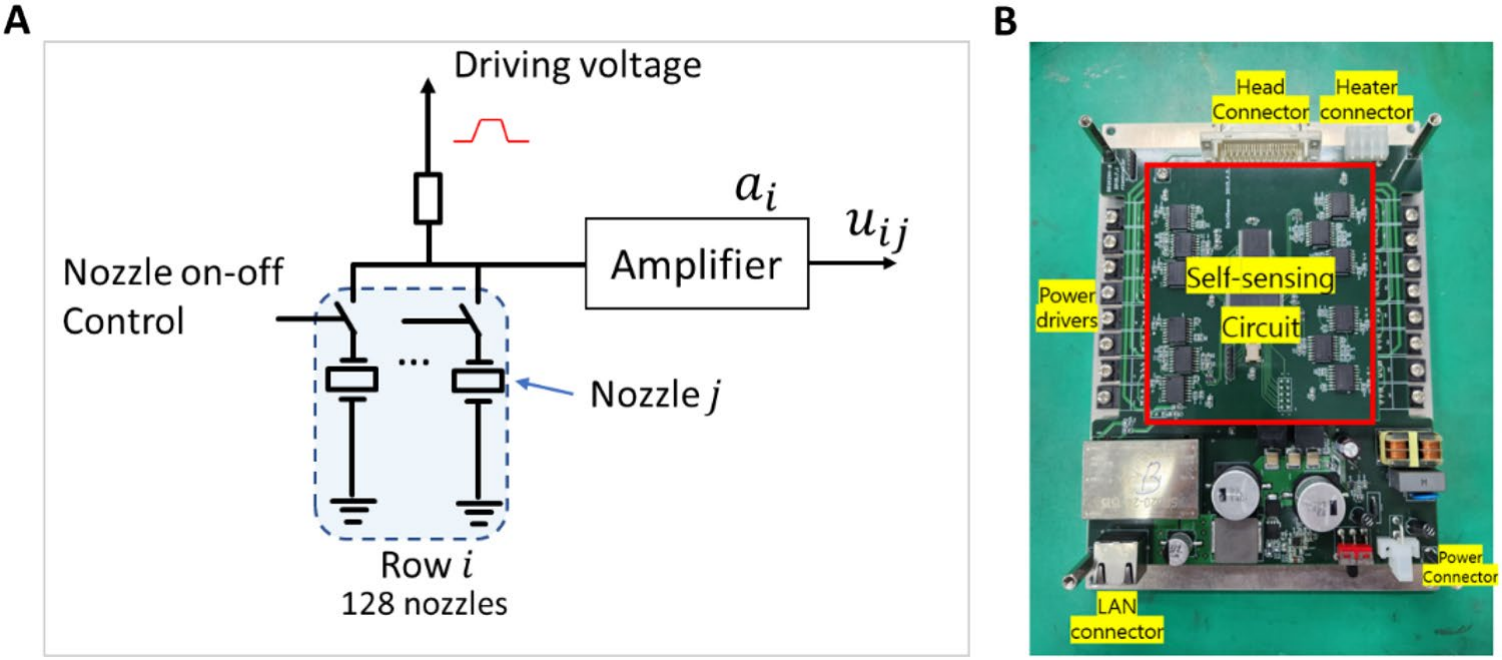
Data acquisition module for self-sensing (**A**) Driving schemes for inkjet, and (**B**) FPGA module for self-sensing signals acquisition.

Figure 4A describes the driving scheme for jetting control and self-sensing in this study. To drive the inkjet head, an electronic power driver applies a waveform to a group of nozzles (one row, 128 nozzles). At the same time, the on–off digital signals are also used to select the jetting nozzles. When a nozzle is selected for printing, it is set to the on-state at the printing position so that the driving waveform can be applied to the corresponding piezo-actuator. Once a driving waveform is applied to a nozzle, the piezo-actuator vibrates and creates a pressure wave for jetting. Since the pressure wave behavior also affects the sensing signal from the piezo, this signal can be measured to monitor the jetting behavior^23^. In this case, the signal from only one nozzle is sensed at a time for monitoring. A scanning scenario developed in our previous study was used to maximize sensing efficiency^22^.

Consider the nozzle $j$ on rows $i$ (for rows $i=1,..,8$, nozzle order $j=1,\dots ,128$), the acquired signal can be written as:

4 $${u}_{ij}={a}_{i}*\left({s}_{ij}+{d}_{ij}+{n}_{ij}\right),$$

where ${a}_{i}$ is the driving voltage amplifier’s characteristics, ${s}_{ij}$ is the self-sensing signal, ${d}_{ij}$ is the influence of the driving signal, and ${n}_{ij}$ is the random noise. The influence of the driving signals can be investigated by measuring the signals when the entire nozzles are off, or ${s}_{ij}\approx 0$. Then, the measured signals do not contain pressure wave information as:

5 $${u}_{ij}^{off}={a}_{i}*\left({d}_{ij}^{off}+{n}_{ij}^{off}\right),$$

where ${d}_{ij}^{off}$ and ${n}_{ij}^{off}$ are the influence of the driving signal and random noise in the off-state. Assume that ${d}_{ij}\approx {d}_{ij}^{off}$, we could remove the driving signal from the data by comparing the two signals:

6 $${v}_{ij}={u}_{ij}-{u}_{ij}^{off}={a}_{i}*\left({s}_{ij}+{n}_{ij}{\prime}\right),$$

with ${v}_{ij}$ being the final signal for analysis. Since ${u}_{ij}^{off}$ helps to remove the influence of the driving signal, it can be considered as nominal data of the nozzle, and the process of removing the nominal data (Eq. 6) is called normalization in this research. For printing, eight drivers are used to control eight rows of nozzle arrays in the printhead. In the case of sensing signals, two driver circuits are combined into a single sensing module, i.e. rows 1 and 2 are paired into a single sensing module, the same with rows 3, 4, and so on. Therefore, the measurement circuit was designed to eliminate the driving voltages^22,23^ so that the effect of the driving voltages ${d}_{ij}$ and ${d}_{ij}^{off}$ could be much smaller than the actual values. However, the influences of driving signals were still significantly high. Therefore, the signals were further removed according to Eq. (6) to obtain more informative self-sensing signals. Then, the self-sensing signals were filtered within the frequency range of 80–400 kHz to suppress the noise and enhance the information about the jetting nozzle. Sensing data were collected by a FPGA module with a sampling rate of 1 MHz, as shown in Fig. 4B.

Accordingly, features were determined by comparing the normalized jetting signal of each nozzle (${v}_{i}$) with a reference signal, ${v}_{i,ref}$. The objective here is to detect significant deviations in jet behavior from the reference status. Note that the initial reference status is the most important, as the judgment of jet status is based on it.

Note that different features can be chosen as long as the jetting information is conveyed^25^. In this study, we used two simple features: the signal phase differences (or phase score – ${\mathrm{\Delta \Phi }}_{i}$) and signal amplitude differences (or amp. score – $\Delta {V}_{i}$) of a nozzle, *i*, in comparison with the corresponding reference signals as:

7 $${\mathrm{\Delta \Phi }}_{i}=\mathrm{acos}\left(\frac{{v}_{i}\cdot {v}_{i,ref}}{\sqrt{{v}_{i}^{2}{v}_{iref}^{2}}}\right)\left(\mathrm{rad}\right),$$

8 $${\Delta V}_{i}=\sum_{k}\left|{v}_{i}\left[k\right]-{v}_{i,ref}\left[k\right]\right| \left(\mathrm{V}\right).$$

Here, $k$ is the sampling time index. Consequently, we denote ${\varvec{x}}$ as the input vector for the model, and the input of an individual nozzle $i$ included its scores:

9 $${{\varvec{x}}}_{{\varvec{i}}}={\left[\Delta {\Phi }_{i},\Delta {V}_{i}\right]}^{T}$$

### Machine learning approaches

The self-sensing signal of normal jetting nozzles is close to its reference signals. In such cases, the scores in Eqs. (7) and (8) are close to zeros. To classify the nozzle jetting status, a boundary must be determined to separate the scores of jetting nozzles from those of non-jetting nozzles in the feature space. In this study, machine learning was used to improve the classification process, allowing the use of more additional features, or can be generalized when scatter plots are not available.

**Figure 5:**
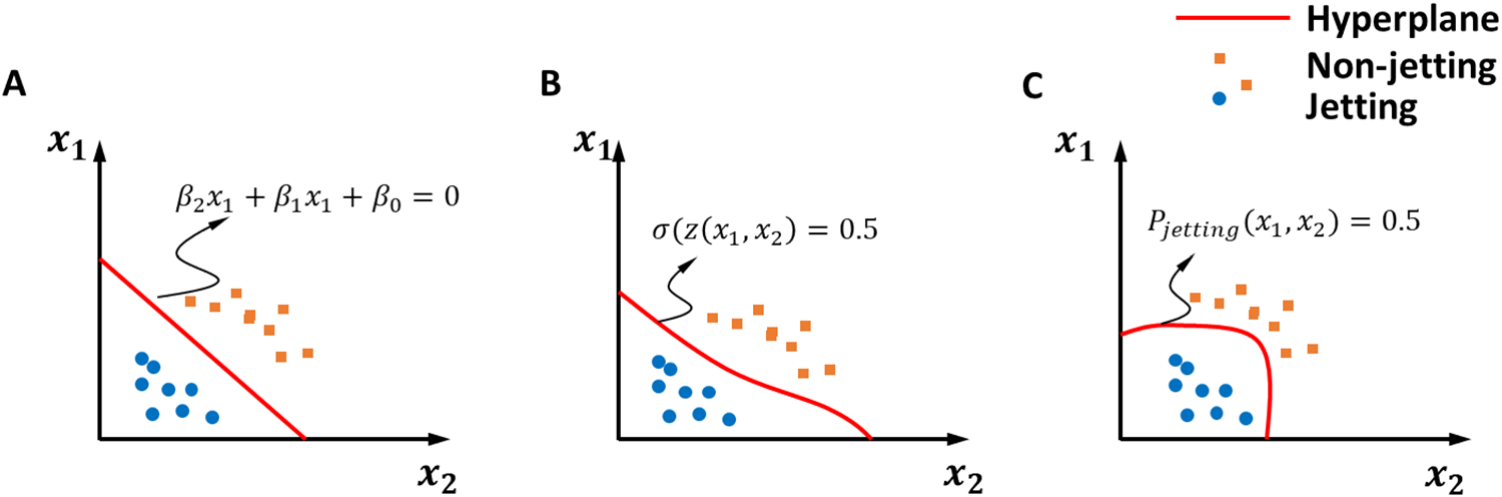
Jetting nozzle detection using the classification of features extracted from self-sensing data. (**A**) Linear support vector machine, (**B**) multilayer neural network, and (**C**) naïve Bayes Gaussian method.

Here, two features are used for the modeling process (phase score and amp. score), and the modeling process becomes a 2D classification problem. Three popular classification models were investigated as shown in Fig. 5. The models determine the hyperplanes to separate regions of failure (non-jetting) nozzles and good (jetting) nozzles in the feature space. The problem can be simplified by assuming that the features have a linear relationship, and a linear support vector machine was used. Additionally, a multilayer neural network was used to compensate for the non-linearity of classification. Furthermore, considering that the features can be independent of each other, the Gaussian naïve Bayes method was also investigated. Table 1 shows the basic parameters of the ML methods used in this study. Note that the Gaussian naïve Bayes method requires no parameters because only simple calculations are performed on the data. The details of the methods can be referred to the Supplementary Information (Sect. S2).

**Table 1:** Customized parameters of the machine learning algorithms.

Method	Parameters
Linear support vector machine	+ Regulation constant C = 1
Multilayer neural network	+ Input: 2 × 1 + Hidden layers: 2 layers Layer 1:100 nodes Layer 2:20 nodes + Active function: sigmoid + Loss function: log-loss

## Results and discussion

### Acquisition and pre-processing of jetting images and self-sensing signals

**Figure 6:**
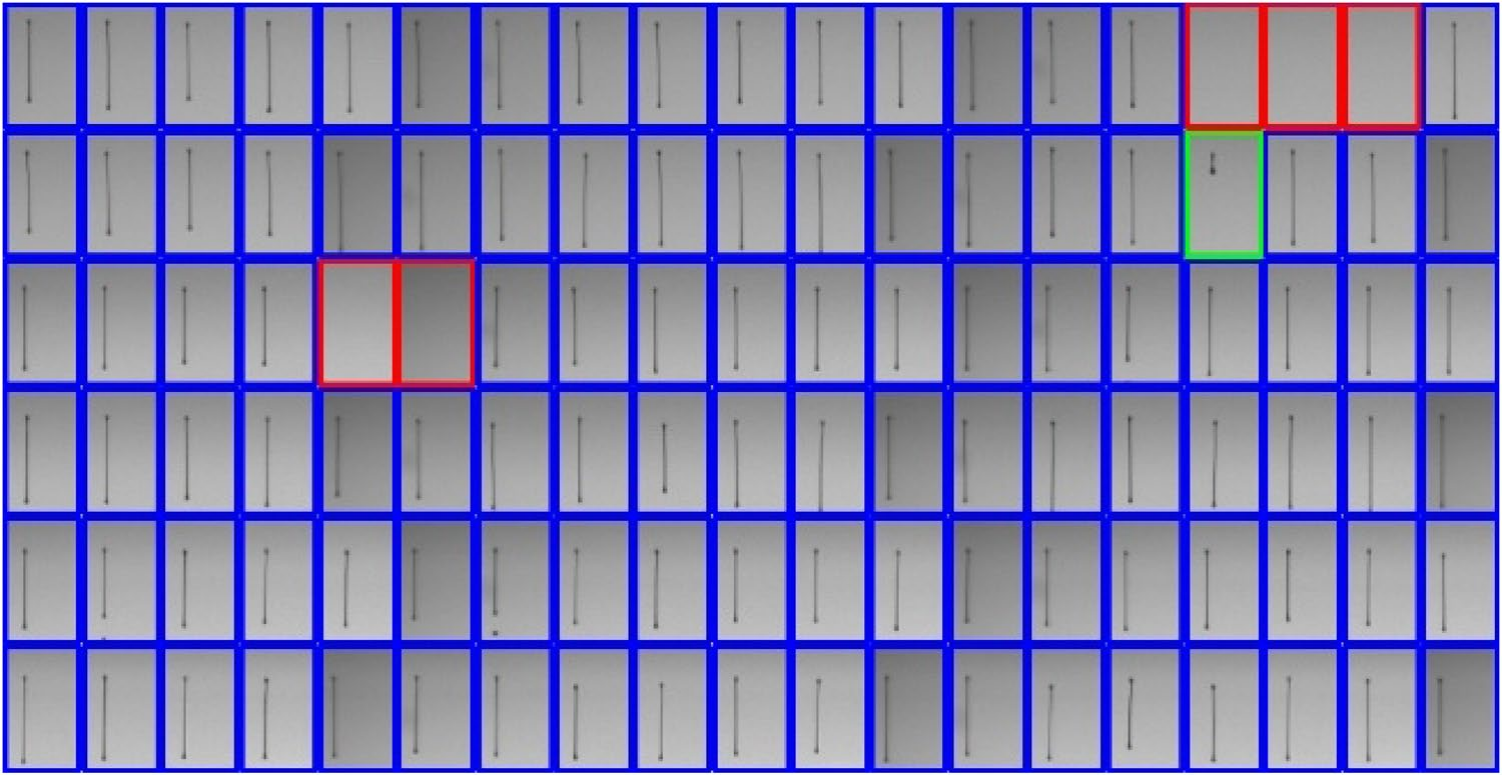
Determination of jetting status with the developed image processing method. (Blue: Jetting, Red: Non-jetting, Green: Slow jetting).

Figure 6 shows an example of determining the jetting status by the developed algorithm. However, in data sampling experiments, poor jetting conditions such as low speed or poor jet direction are rarely observed. Therefore, in this study, only two jetting states were considered for self-sensing analysis: the (good) jetting and non-jetting status. For simplicity, we considered slow jetting as the (good) jetting status. Accordingly, the jetting status of each nozzle, ${y}_{i}$, is quantified for calculation as:

10 $$y_{i} = \left\{ {\begin{array}{*{20}c} {1,} & {jetting} \\ { - 1,} & {non - jetting} \\ \end{array} } \right.$$

The features ${{\varvec{x}}}_{i}$ (Eq. (9)) and its corresponding label ${y}_{i}$ (Eq. (10)) were used as the data for the training and verification of the models.

**Figure 7:**
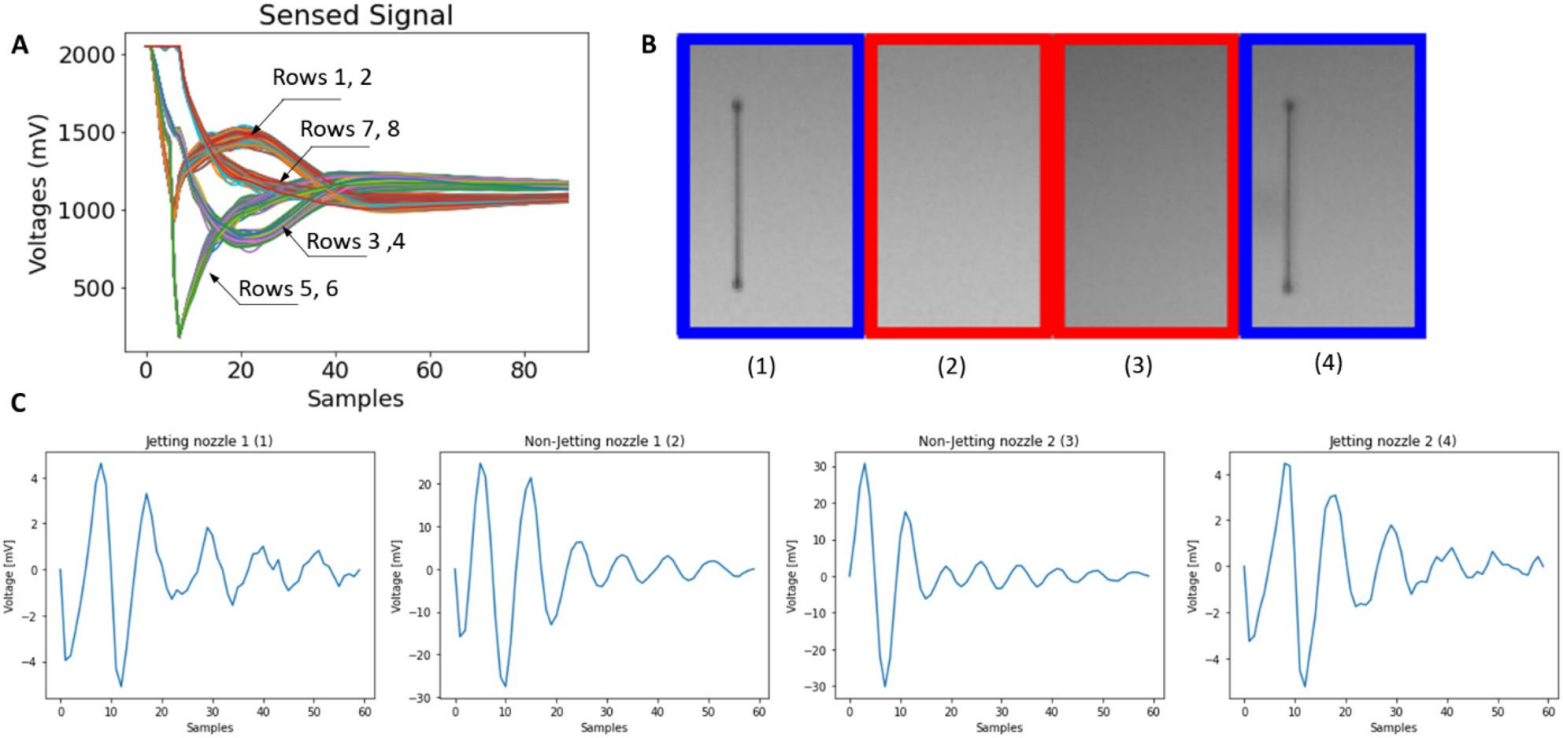
Acquired self-sensing signals. (**A**) Original self-sensing signals of all nozzles (**B**) Samples of consecutive nozzles with different conditions in row 4, and (**C**) pre-processed signals of the nozzles in (**B**).

**Figure 8:**
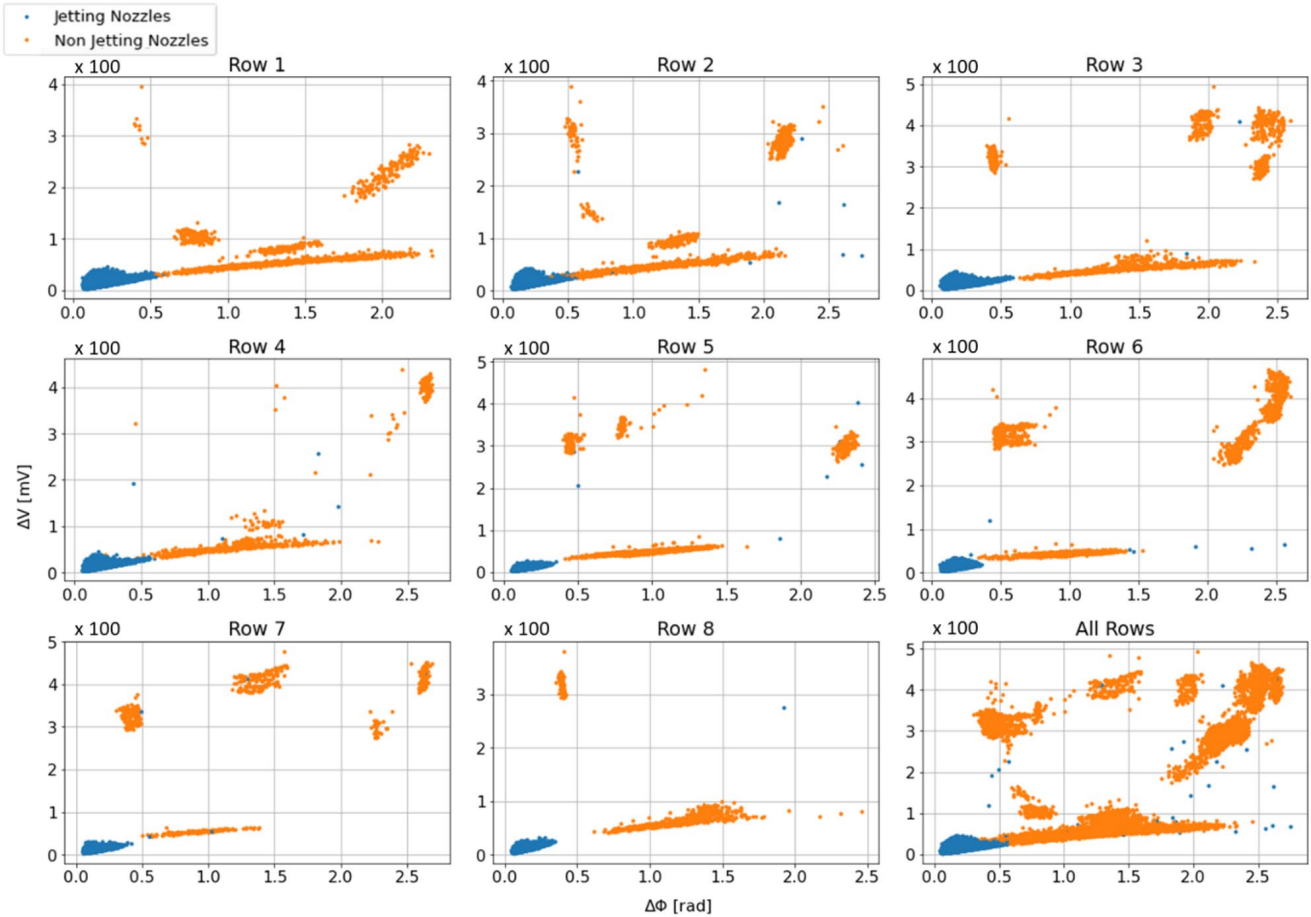
Data for modelling of jetting status. The data was labeled using the developed image processing method.

Figure 7 shows the acquired self-sensing signals. Since the rows are controlled by different controllers, the raw acquired signals vary from row to row as shown in Fig. 7A. Before feature extraction, the signals should be pre-processed. As shown in Fig. 7B and C, the pre-processed signals acquired from jetting and non-jetting nozzles are quite different in amplitudes and phases. After pre-processing, the features were extracted to have the final data for the modeling process shown in Fig. 8. Note that although the features of jetting nozzles vary according to the nozzle rows (sensing module) (Fig. 8), the row-dependent characteristics were not considered. Here, we aim to build the same model for all the jetting cases in the printhead as well as for different printheads.

### Jetting status modeling

To gain insight into the modeling results, we investigated the performance of model training and trained models. Here, the data (more than 150,000 jetting samples) were shuffled so that 80% was used for training (training data, ~ 120,000 samples), and the rest (test data, ~ 30,000 samples) was used to verify the models.

**Figure 9:**
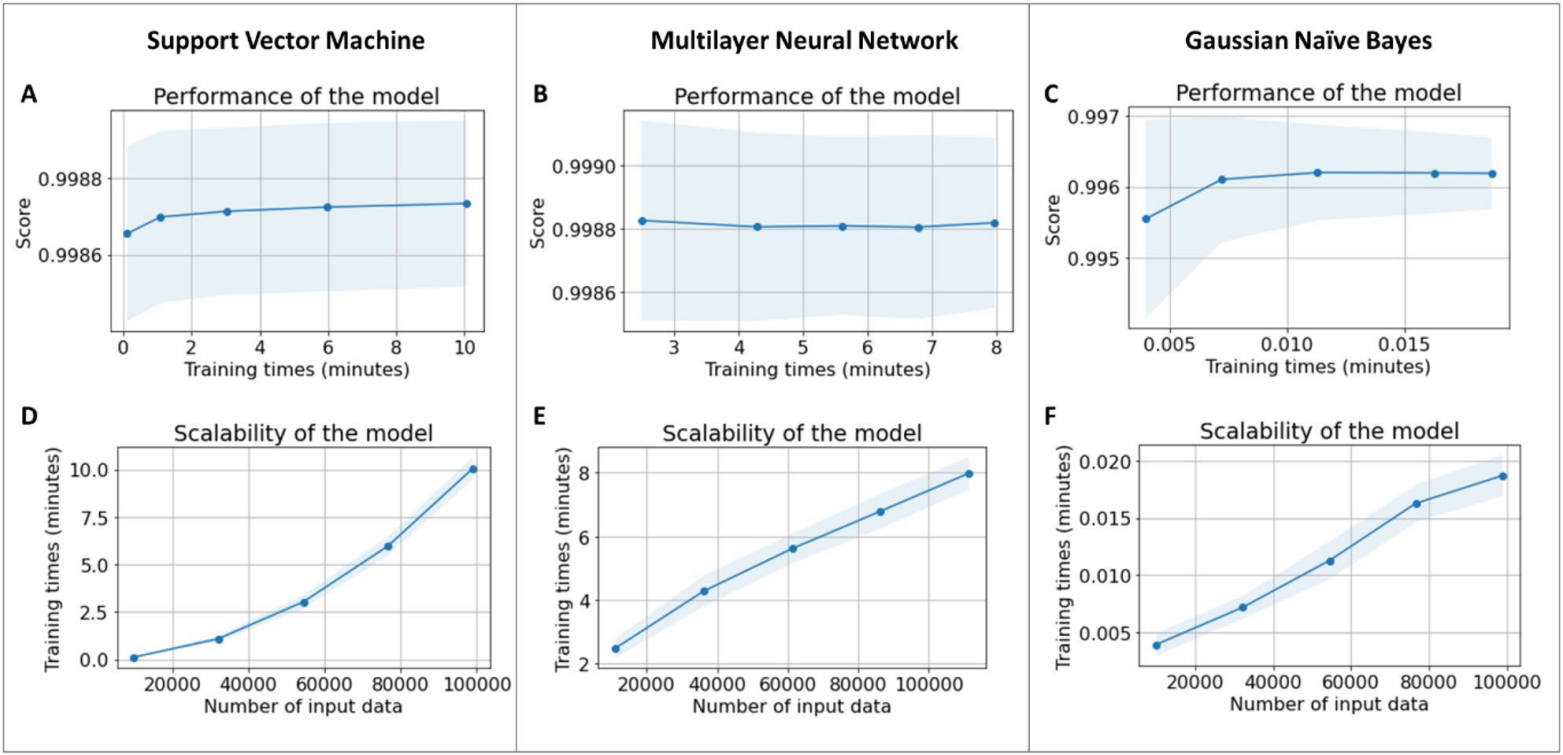
Training performance of classification models. (**A**,**D**) Support vector machine, (**B**,**E**) Multilayer neural network, and (**C**,**F**) Gaussian naïve Bayes. The shade shows the standard deviation values.

Figure 9 shows the performance of the models trained using the same training equipment and training. As shown in Fig. 9, all three models provided an accuracy of more than 99.6% with respect to the training data. In general, the multilayer neural network model showed the best training performance with the accuracy of about 99.8%. The accuracy of the support vector machine model was slightly lower than the multilayer network. The naïve Bayes model had a slightly less accuracy of around 99.6%. However, Figs. 9D–F show that the naïve Bayes classifier trained 500 times faster than the other two methods because it only needed simple computations and not complex optimization. Compared to the multilayer neural network, the support vector machine trained faster when the number of training data was less than 6,000. However, the multilayer neural network model took less time if the amount of training data increased. Moreover, Fig. 9 shows that the support vector machine model required more data to stabilize accuracy with ~ 6000 training samples, whereas ~ 3000 data samples were sufficient to train the multilayer neural network and Gaussian naïve Bayes models. For details, the metrics of the models after training regarding training data and test data are presented in Table 2.

**Table 2:** Metrics of the models.

		Training data				Test data			
		Precision	Recall	F1-score	Accuracy	Precision	Recall	F1-score	Accuracy
Support vector machine	Non-jetting	0.99536	0.9868	0.99106	0.99878	0.99608	0.98737	0.99171	0.9989
	Jetting	0.99903	0.99966	0.99934		0.9991	0.99972	0.99941	
Multilayer neural network	Non-jetting	0.99094	0.99257	0.99176	0.99887	0.99271	0.99223	0.99247	0.999
	Jetting	0.99945	0.99933	0.99939		0.99945	0.999498	0.99946	
Gaussian naïve Bayes	Non-jetting	0.94891	0.99823	0.97295	0.99619	0.95711	0.99709	0.97669	0.99683
	Jetting	0.99987	0.99604	0.99795		0.99979	0.99681	0.9983	

**Figure 10:**
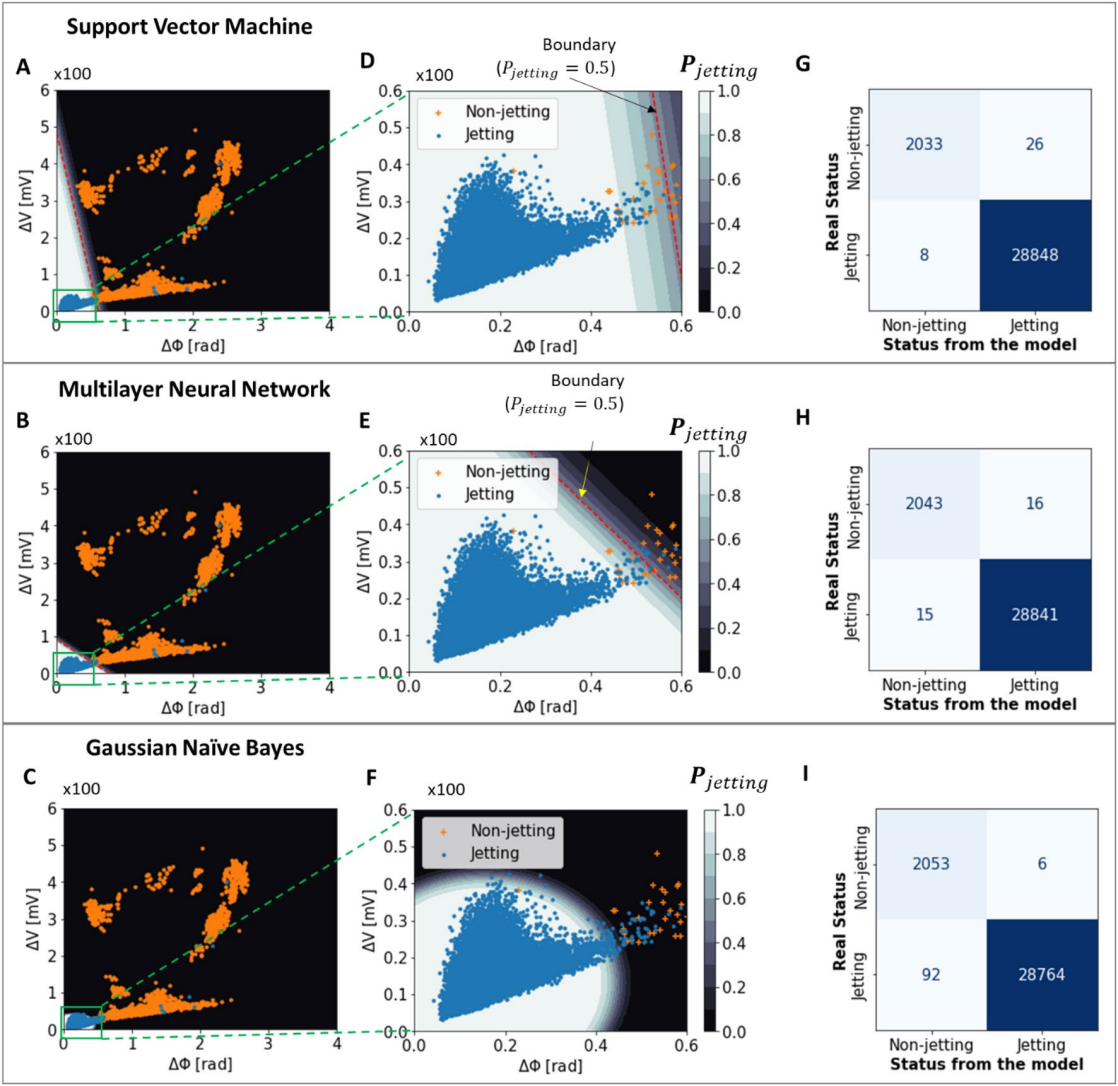
Confusion matrices on test data and classifiers’ probability distributions of classification models.

To better understand and evaluate the trained models, the classification results of the model were examined with the test data, as shown in Fig. 10. Note that the phase and amp. scores of the jetting and non-jetting nozzles overlapped (Figs. 8 and 10), possibly due to electronic noises during the measurement. Moreover, different nozzles have different characteristics, so the features (phase and amp. scores) of some jetting nozzles could be mixed with those of other non-jetting nozzles. Therefore, even though the classification was based on optimal models, there were still misclassified nozzles. Here, there are two types of misclassification: jetting nozzles are classified as non-jetting ones (case 1), and non-jetting nozzles are classified as jetting ones (case 2), as shown in Fig. 10D–F. Note that compared to case 1, case 2 (selecting the wrong nozzles for printing) is more critical in terms of printing quality. On the other hand, if some jetting nozzles are not selected (case 1), only the efficiency of the printing process may be reduced without deteriorating printing quality.

The success rate of nozzle classification is different depending on the models. Figure 10D–F show that the naïve Bayes model provided better results in the selection of jetting nozzles. According to the models' confusion matrices (Fig. 10G–I), the naïve Bayes model had only six cases of non-jetting nozzles that were defined as jetting nozzle (case 2) out of 2059, compared to 26 and 16 cases for the support vector machine and multilayer neural network, respectively. However, among the models, it has the most cases such that jetting nozzles are classified as non-jetting nozzles (92 nozzles of case 1 misclassification), which may reduce the efficiency of the process. Besides, although the support vector machine classifier had significant misclassified nozzles of case 2, it showed the lowest misclassification of case 1. In other words, the support vector machine classifier tries to select as many nozzles as possible, while the naïve Bayes model ensures the selection of jetting nozzles. To ensure the quality of the printing, the naïve Bayes model is preferable. Furthermore, the naïve Bayes model had the fastest training time, which is suitable for online training (acquiring the sensing signals and updating the data simultaneously with training the model) in real-time.

### Prediction probability characteristics and enhancement of prediction performance using hybrid monitoring method

**Figure 11:**
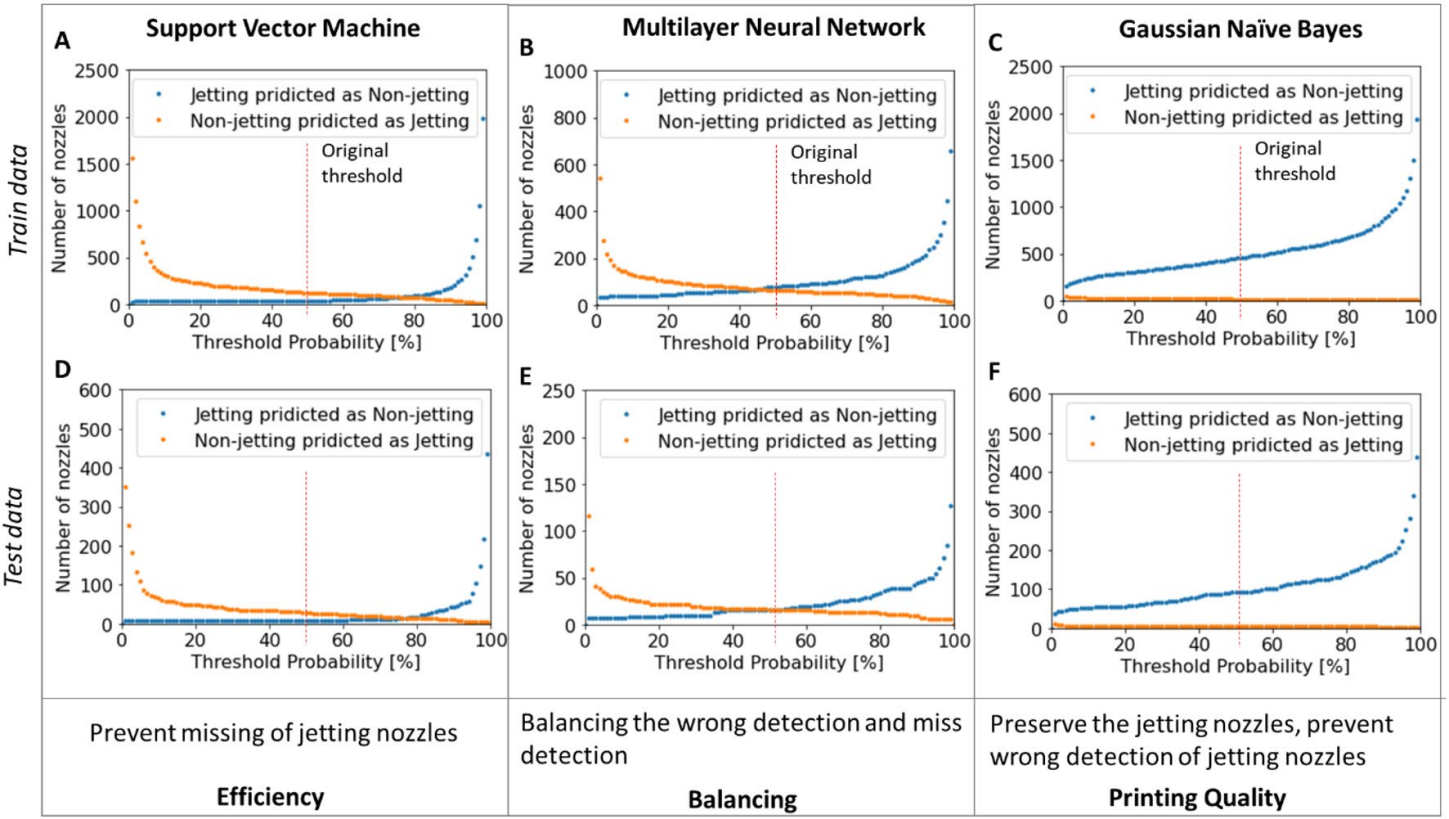
Model characteristics according to the probability of jetting.

To improve classification efficiency, it is necessary to understand how models process the data for classification. Initially, the classifiers used the predicted jetting probability of ${P}_{jetting}$ = 0.5 (50% jetting–50% non-jetting) as the threshold for classifying the nozzle jetting states (Supplementary Information Sect. S2). Figure 11 shows the behavior of the models when the threshold varies.

At the threshold of ${P}_{jetting}$ = 0.5, the naïve Bayes classifier seems to be more reliable for selecting jetting nozzles for printing, as it prevents non-jetting nozzles from being selected as jetting nozzles. On the contrary, the support vector machine classifier can increase printing efficiency by classifying the jetting nozzles as much as possible. However, non-jetting nozzles can be selected as jetting nozzles, which causes printing defects. The multilayer neural network model balances both cases.

**Figure 12:**
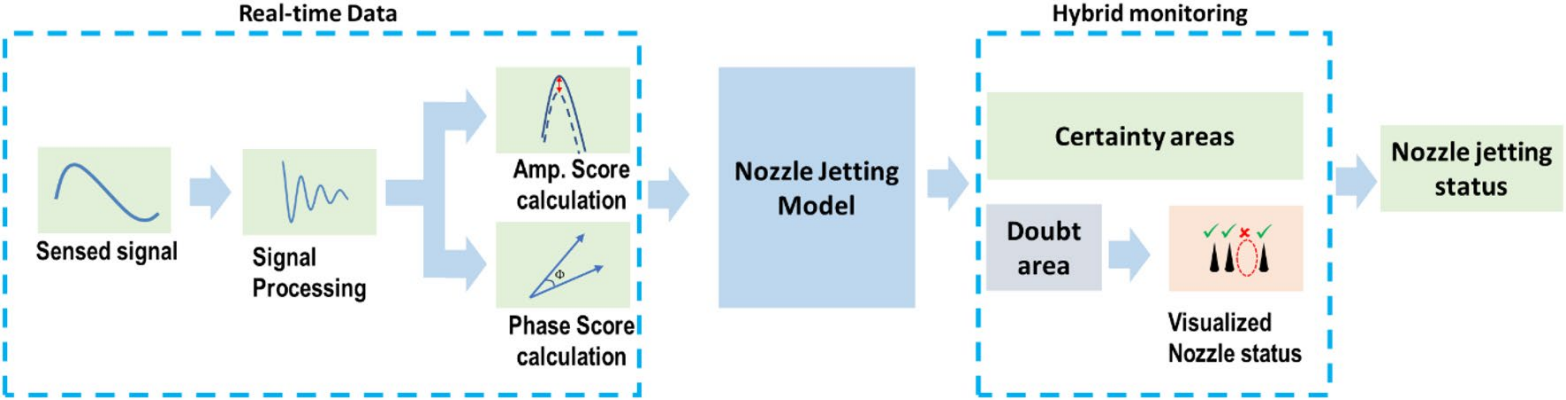
Hybrid monitoring using trained models and visualization.

In all cases, as the predicted jetting probability increases, fewer non-jetting nozzles are classified as jetting nozzles, but more jetting nozzles are misclassified as non-jetting (Fig. 11). In this case, we can customize the predicted jetting probability (${P}_{jetting}$) threshold for our purposes. For instance, the predicted jetting probability threshold can be increased to ensure print quality, or the threshold can be lowered to increase the number of nozzles for printing. In our study, we proposed a hybrid monitoring approach combining self-sensing and jetting monitoring (drop visualization), as described in Fig. 12, to reduce the incorrect selection of non-jetting nozzles for printing and improve printing efficiency.

**Figure 13:**
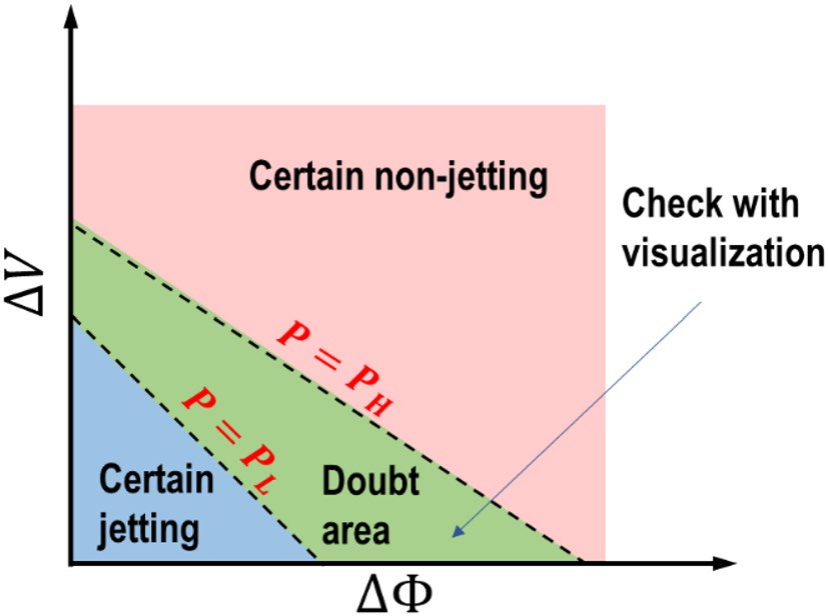
The use of two predicted jetting probability thresholds for hybrid monitoring.

In this method, a decision boundary with a single probability threshold is not used. Instead, we used two values, ${P}_{H}$ and ${P}_{L}$, for thresholding the predicted jetting probability of the models to divide the feature space into three regions as shown in Fig. 13:

11 $$\left\{ {\begin{array}{*{20}c} {P_{{jetting}} \left( {x_{i} } \right) \ge P_{H} ,} & {certain\,\,jetting} \\ {P_{{jetting}} \left( {x_{i} } \right) < P_{L} ,} & {certain\,\,non - jetting} \\ {P_{H} > P_{{jetting}} \left( {x_{i} } \right) \ge P_{L} ,} & {Doubt\,\,\,area} \\ \end{array} } \right.$$

Here, the first high probability (${P}_{H}$) was used as a strict threshold to define a certain jetting region (for example, the predicted probability of jetting is greater than 95%). All the nozzles in this region are classified as jetting nozzles, and no additional jetting verification is required. Another lower probability threshold (${P}_{L}$) was used to determine non-jetting nozzles. This threshold may not be strict. The actual jetting nozzles in this region can be excluded without affecting printing quality. The nozzles in the region of doubt, ${P}_{H}\ge {P}_{jetting}>{P}_{L}$, can be examined by a drop visualization (or test printing patterns) to confirm the jetting status. Note that the selection of ${P}_{L}$ and ${P}_{H}$ depends on the required level of rigor and printing efficiency.

**Figure 14:**
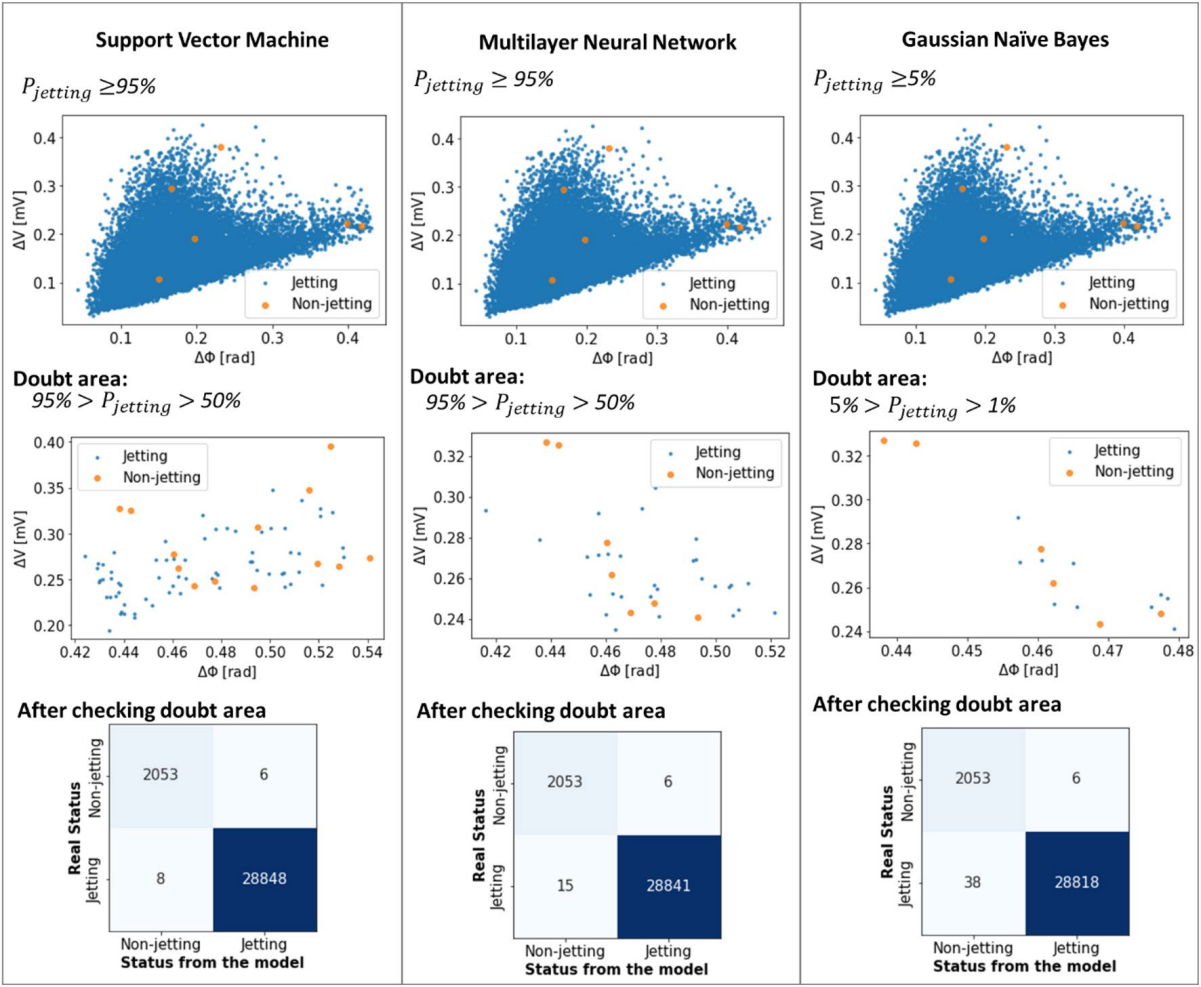
Hybrid monitoring methods for verifying nozzle status using two probability thresholds.

Figure 14 describes the instances of the doubt region for the models. The recommended thresholds are 1–5%, 50–95%, and 50–90% for the naïve Bayes, support vector machine, and multilayer neural network, respectively in this study. As shown in Fig. 14, in this study, if the doubt regions were verified, the number of missed jetting nozzles (case 1) in the Gaussian naïve Bayes model reduced more than two times (from 92 to 38 cases) compared to the single threshold for probability discussed earlier in Fig. 10. Similarly, the number of misclassified non-jetting nozzles (case 2) also reduced more than 3 and 2.5 times, from 26 and 16 to 6 cases, in the cases of support vector machine and multilayer neural network models respectively.

As shown in Fig. 14, the hybrid method had the best fit with the support vector machine model. However, the naïve Bayes model appeared stable in determining the jetting nozzles for printing (Figs. 11 and 14). Although the lower jetting threshold (${P}_{L}$) was reduced to 1%, and a few non-jetting nozzles appeared in the jetting region. Therefore, when only the ML model is used without additional verification (visual or print inspection) for the selection of jetting nozzles, the naïve Bayes classifier should be chosen. According to Figs. 10 and 14, we can observe outliers in the jetting regions and non-jetting regions. The scores of some non-jetting nozzles (or jetting nozzles) are mixed with those of jetting nozzles (non-jetting nozzles), so the training performance and verification cannot reach 100%. This may be due to the reliability of the nozzles, and the asynchrony of visualization and self-sensing during data collection for modeling. However, because the models were trained using very sparse error data, these outliers do not affect their generalizability.

## Conclusion and future perspectives

In this study, we proposed the use of machine learning models to monitor the jetting status of multi-nozzle printheads (with more than 1000 nozzles) using self-sensing signals. The purpose is to automate the detection of the nozzles experiencing jet failure (non-jetting nozzles) and subsequently eliminate them from the printing process.

For this purpose, significant parameters (here, magnitude and phase differences) were derived by extracting the self-sensing signals of the piezo actuators of the inkjet heads. To label and subsequently verify the condition of the nozzles, an improved algorithm was developed for batch droplet image processing. Three ML models, including the support vector machine, the multilayer neural network, and Gaussian naïve Bayes, were investigated, yielding accuracies of more than 99.6%. Here, the Gaussian naïve Bayes model is preferred due to its fastest training and higher reliability. This model exhibits fewer misclassifications of non-jetting nozzles, an important factor in ensuring print quality.

To enhance the accuracy of nozzle status determination, we introduced a combination of self-sensing detection and drop visualization for monitoring. Two thresholds for the probabilities of jetting detection were employed, allowing the categorization of nozzle status into three regions: certain jetting, certain non-jetting, and a doubt region. Within this doubt region, nozzle conditions can be verified through drop visualization (or printing) inspection, reducing misclassifications and improving printing performance.

While the machine learning approach proves applicable to the monitoring process, it comes with certain limitations. Specifically, the results remain dependent on features extracted from signals, making them susceptible to noise and pre-processing parameters. This constraint hinders the detection of additional types of nozzle defects. For future perspective, the development of AI techniques, such as Convolutional Neural Networks (CNN) or Recurrent Neural Networks (RNN) for self-sensing signals, holds promise in bypassing pre-processing steps and enhancing the real-time and accurate detection of faulty jet behaviors.

### Supplementary Information

Supplementary Information.

## Data Availability

The datasets generated and/or analyzed during the current study are available from the corresponding author upon reasonable request.
