# Supplementary material for: Machine learning approach to monitor inkjet jetting status based on the piezo self-sensing
Source: Sci Rep. 2023 Oct 23;13:18089. doi: 10.1038/s41598-023-45445-0 (PMC10593807; doi:10.1038/s41598-023-45445-0)
Supplement: Supplementary file 1 — Supplementary Information. [file 41598_2023_45445_MOESM1_ESM.docx]

**Supplementary Information**

Machine learning approach to monitor inkjet jetting status based on the piezo self-sensing

Thanh Huy Phung^1,2^, Sang Hyeon Park^3^, Inyoung Kim^4,5^, Taik-Min Lee^4,5,**^, and Kye-Si Kwon^,3,6*^

^1^ Department of Mechatronics, Ho Chi Minh University of Technology (HCMUT), 268 Ly Thuong Kiet Street, District 10, Ho Chi Minh City 700000, Vietnam

^2^ Vietnam National University Ho Chi Minh City (VNU-HCM), Linh Trung Ward, Thu Duc, Ho Chi Minh City 700000, Vietnam

^3^ Department of Electronic Materials Devices and Equipment Engineering, Soonchunhyang University, 22 Soonchunhyang-ro, Shinchang, Asan-si, Chungnam, 31538, Republic of Korea

^4^ Department of Flexible and Printed Electronics, Korea Institute of Machinery and Materials (KIMM), 156 Gajeongbuk-ro, Yuseong-gu, Daejeon 34103, Republic of Korea

^5^ Department of Robot and Manufacturing System, Korea University of Science and Technology (UST), 217 Gajeong-ro, Yuseong-gu, Daejeon, 34113, Republic of Korea

^6^ Department of Mechanical Engineering, Soonchunhyang University, 22 Soonchunhyang-Ro, Shinchang, Asan-si, Chungnam, 31538, Republic of Korea

**S1. Jetting status classification using gradient-based image processing**

As mentioned in the main text (Section 2.3), the frozen jetting images should be preprocessed to calculate the image gradients. After calculating the gradients (Eq. 6, main text), the peaks can be determined as shown in Fig. 3 (main text). These peaks can be used to determine almost all the jetting conditions of the nozzles.

Figure S1 illustrates the proposed method to determine the jetting status based on the gradient peaks. As described in Fig. S1, the number of positive peaks in $D_{1}$ represents the number of droplet segments (including satellites), and a segment length can be calculated by the distance from the positive peak to its consecutive negative peak. The total jetting length (the whole droplet) can be calculated from the distance from the first positive peak to the last negative peak.


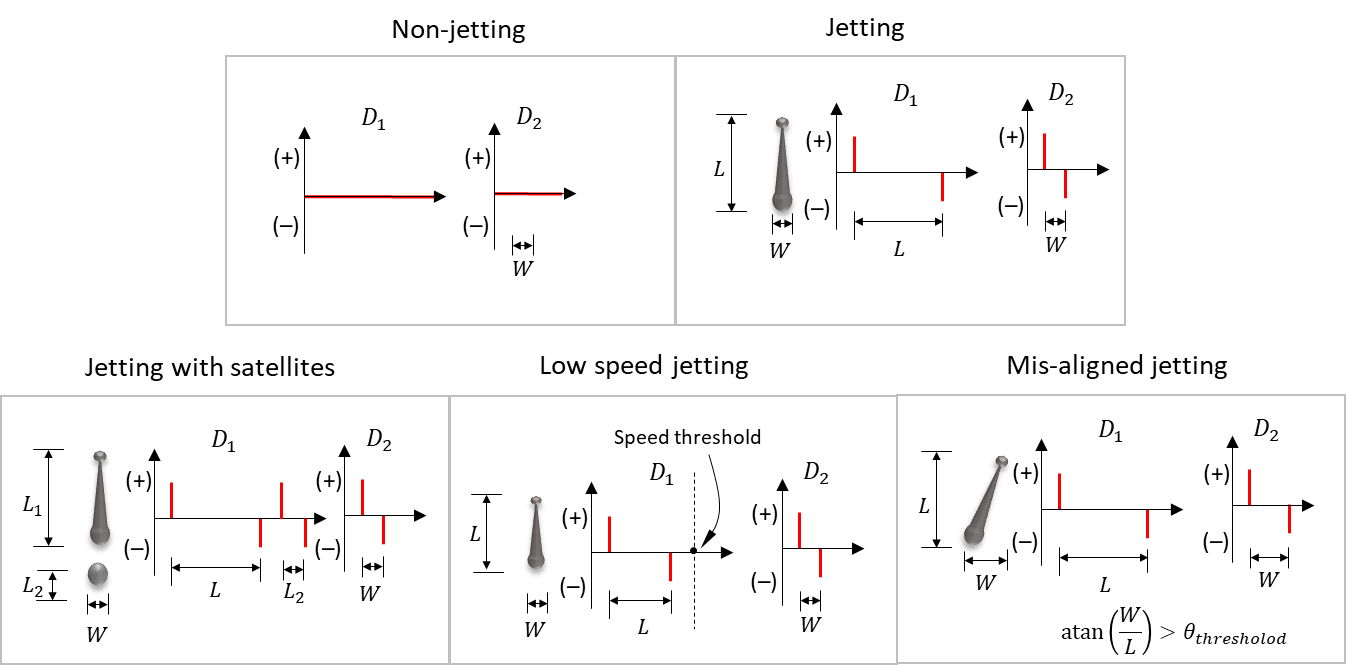


**Figure S1.** Jetting status classification by using peaks in image gradients.

Accordingly, there are no ligaments in the case of non-jetting, so no peaks can be found in both $D_{1}$ and $D_{2}$ (Eq. (6), main text). In normal jetting, $D_{1}$ and $D_{2}$have only 1 positive peak, which can be distinguished from the jetting nozzles that have satellites ($D_{1}$, $D_{2}$have more than 1 positive peak) and slow jetting speed (the total jetting length is shorter than a threshold level). Moreover, the distance from the first positive peak to the last negative peak at $D_{2}$ shows the horizontal projection of the droplet image. If the distance between the positive and negative peaks in $D_{2}$ is greater than some threshold, the jetting can be defined as misdirected (jet direction with non-zero angles).

**S2. Machine learning approaches to determine nozzle status**

In this study, the jetting conditions were classified into jetting and non-jetting status based on the features calculated from the self-sensing signals (phase score and amp. score) as:

$\boldsymbol{x}_{\boldsymbol{i}}=\left[ \Delta\Phi_{i}, \Delta V_{i} \right]^{T}$ (S1)

For this purpose, the jetting status was numerically described as:

$y_{i}=\left\{ \begin{aligned} 1, jetting \\ -1, non-jetting \end{aligned} \right.$ (S2)

Three popular classification algorithms, including support vector machine, multilayer neural network, and Gaussian naïve Bayes, were investigated. This section describes the method selection and the details of the methods.

#### Support vector machine

As shown in Fig. 8 (Main text), the scores of jetting nozzles and non-jetting nozzles are quite separable. Therefore, a straight line can be used to divide the data in the scoring space: the part below the line (lower scores) for jetting nozzles, and the part above the line (higher scores) for non-jetting nozzles. For this purpose, a popular linear support vector machine classifier can be used. In this method, the line separator between jetting and non-jetting nozzles has a linear form:

$\boldsymbol{\beta}^{\boldsymbol{T}}\boldsymbol{x}+\beta_{0}=0$ (S3)

In our case, two features ($\Delta V$ and $\Delta\Phi$) were used, the Eq. (S3) represents a line. If the number of features increases, Eq. (S3) becomes a hyperplane. The coefficient $\boldsymbol{\beta}$ and intercept $\beta_{0}$ of the separator are determined by ^S1^:

$\left( \boldsymbol{\beta},\beta_{0} \right)=\arg\min_{\left( \beta,\beta_{0} \right)} \left( \frac{1}{2}\boldsymbol{\beta}^{T}\boldsymbol{\beta}+C\sum_{i} \max\left\{ 0,1-y_{i}\left( \boldsymbol{\beta}^{T}\boldsymbol{x}_{\boldsymbol{i}}+\beta_{0} \right) \right\} \right)$ (S4)

Here, the constant $C>0$ is a factor that balances regulation and performance during training. In this study, *C* = 1 was used. For later uses, the jetting possibility could be calculated by Platt’s calibrator ^S2,S3^:

$P_{jetting}\left( \boldsymbol{x}_{i} \right)=\frac{1}{1+\exp\left( a\left( \boldsymbol{\beta}^{T}\boldsymbol{x}_{\boldsymbol{i}}+\beta_{0} \right)+b \right)}$ (S5)

where constants $a \mathrm{and} b$ can be calculated from the model such that the linear separator in Eq. (S3) has a jetting probability of $P_{jetting}=0.5$.

#### Multilayer Neural Network

Note that the support vector machine method is based on a linear separator. In this section, we will use a multilayer neural network classifier to compensate for the nonlinearity relationship between phase and amp. scores of the nozzles. Since the processed data is highly separable (Fig. 10, Main text), a deep neural network (a network with lots of layers) may not be required, a shallow neural network was considered as described in Fig. S2.

*
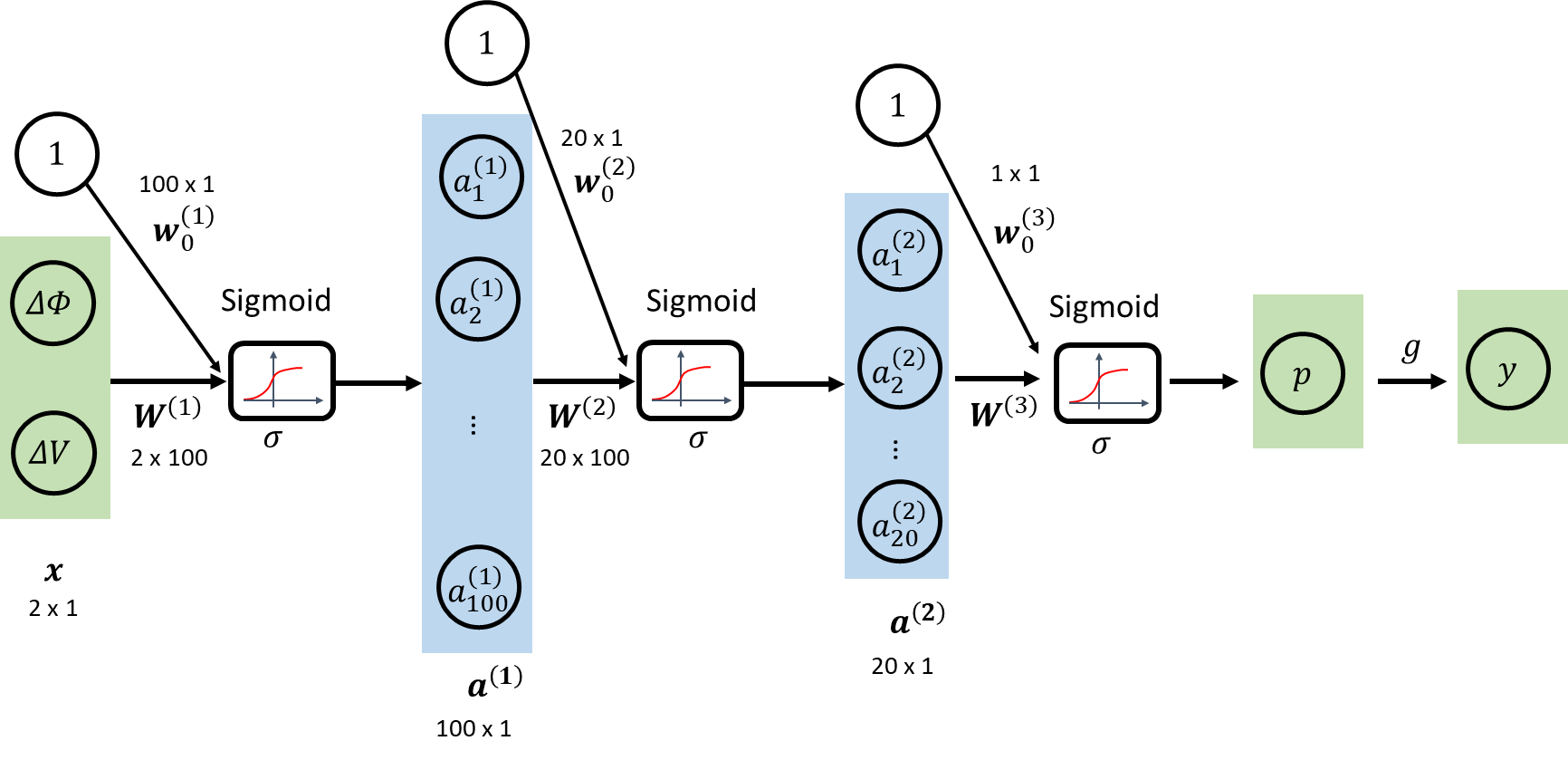
*

**Figure S2**. Multilayer neural network structure for the classification of the nozzle status. The superscripts in parentheses indicate the corresponding layer.

The working of the network in Fig. S2 could be mathematically described as follows:

$\left\{ \begin{aligned} \boldsymbol{a}^{(1)}=\sigma\left( {\boldsymbol{W}^{\left( 1 \right)}}^{T}\boldsymbol{x}+\boldsymbol{w}_{0}^{\left( 1 \right)} \right) \\ \boldsymbol{a}^{\left( 2 \right)}=\sigma\left( {\boldsymbol{W}^{\left( 2 \right)}}^{T}\boldsymbol{a}^{\left( 1 \right)}+\boldsymbol{w}_{0}^{\left( 2 \right)} \right) \\ p=\sigma\left( {\boldsymbol{W}^{\left( 3 \right)}}^{T}\boldsymbol{a}^{\left( 2 \right)}+\boldsymbol{w}_{0}^{\left( 3 \right)} \right) \\ \hat{y}=g\left( p \right) \end{aligned} \right.$ (S6)

where $\sigma$ is the activation function. Since we classified 2 groups (jetting and non-jetting), the logistic (sigmoid) activation function was used as follows:

$\sigma\left( z \right)=\frac{1}{1+e^{-z}}$ (S7)

Since $\lim_{z\to\infty} \sigma=1$and $\lim_{z\to-\infty} \sigma=0$, $p_{i}$ could be considered as the predicted probability of the nozzle jetting status. Accordingly, the predicted jetting status of the nozzle $i$:

$\hat{y}_{i}=g\left( p_{i} \right)=\left\{ \begin{aligned} Jetting, p_{i}\geq0.5 \\ Non-jetting, p_{i}<0.5 \end{aligned} \right.$ (S8)

and the boundary between jetting and non-jetting nozzles is at $P_{jetting}=p=$0.5. The coefficients $\boldsymbol{W}^{(1)},\boldsymbol{W}^{\left( 2 \right)},\boldsymbol{W}^{\left( 3 \right)}$ and intercepts$\boldsymbol{w}_{0}^{(1)},\boldsymbol{w}_{0}^{\left( 2 \right)},\boldsymbol{w}_{0}^{\left( 3 \right)}$ could be calculated by minimizing the total log-loss function of all training data ^S1,S4^:

$L_{log}\left( y,p \right)=-\sum_{i} \left( y_{i}\log p_{i}+\left( 1-y_{i} \right)\log\left( 1-p_{i} \right) \right)$ (S9)

#### Gaussian Naïve Bayes Model

In the support vector machine and multilayer neural network, features (phase and amp. scores) seem to have binding relationships. Moreover, since the two former methods are based on the optimization of the Eqs. (S4), and (S9), the training time and the number of training data samples are considerable. Therefore, we attempted to develop another, simpler model with the following assumptions: the influences of phase and amp. scores are independent, i.e. the abnormality in either phase or amplitude may be indicative of a failure nozzle. For this purpose, the Gaussian Naïve Bayes Model was used. As a result, the jetting probability based on the Gaussian naïve Bayes of the independent variables is ^S1^:

$P_{jetting}\left( \boldsymbol{x}_{\boldsymbol{i}} \right)\triangleq P\left( y_{i} | \Delta\Phi_{i}, \Delta V_{i} \right)=\frac{P\left( y_{i} \right)P\left( \Delta\Phi_{i} | y_{i} \right)P\left( \Delta V_{i} | y_{i} \right)}{P\left( \Delta\Phi_{i})P( \Delta V_{i} \right)}$ (S10)

$P\left( y_{i} \right)$is calculated through the appearance of the jetting nozzle in the dataset. The margin probability $P\left( \Delta\Phi_{i},\Delta V_{i} \right)$ is calculated by assumming that $\Delta\Phi_{i}$, $\Delta V_{i}$ are independent and Gaussian, or:

$P\left( \Delta\Phi_{i},\Delta V_{i} \right)=P\left( \Delta\Phi_{i})P(\Delta V_{i} \right)$ (S11)

$P\left( \Delta\Phi_{i} \right)=\frac{1}{\sqrt{2\pi\sigma_{\Phi}^{2}}}\exp\left( -\frac{({\Delta\Phi}_{i}-\mu_{\Phi})^{2}}{2\sigma_{\Phi}^{2}} \right)$ (S12)

where $\sigma_{\Phi}^{2}, \mu_{\Phi}$ are the variance and means of $\Delta\Phi_{i}$ in the dataset. The same is applied for $P\left( \Delta V_{i} \right)$

Similarly, the jetting probability regarding each feature is calculated by assuming that the features are Gaussian, or:

$P({\Delta\Phi}_{i}|y_{i})=\frac{1}{\sqrt{2\pi\sigma_{\Phi y}^{2}}}\exp\left( -\frac{({\Delta\Phi}_{i}-\mu_{\Phi y})^{2}}{2\sigma_{\Phi y}^{2}} \right)$ (S13)

where $\sigma_{\Phi y}^{2}, \mu_{\Phi y}$ are the variance and means of the features from the jetting nozzles. The same applies for $P\left( \Delta V_{i} | y_{i} \right)$.

The boundary between non-jetting and jetting nozzles is also at $P_{jetting}$= 0.5.

**References**

S1. Hastie, T., Tibshirani, R., James, G. & Witten, D. An introduction to statistical learning (2nd ed.). *Springer texts* **102**, 618 (2021).

S2. John C. Platt. Probablistic outputs for svm and comparison to regularized likelihood methods. *Adv. Large Margin Classif.* **10**, 61–74 (1999).

S3. Lin, H.-T., Lin, C. & Weng, R. C. A note on Platt’s probabilistic outputs for support vector machines. *Mach. Learn.* **68**, 267–276 (2007).

S4. Buitinck, L. *et al.* API design for machine learning software: experiences from the scikit-learn project. 1–15 (2013).
